# Phospholipase activity of acyloxyacyl hydrolase induces IL‐22‐producing CD1a‐autoreactive T cells in individuals with psoriasis

**DOI:** 10.1002/eji.202149485

**Published:** 2022-01-10

**Authors:** Randeep Singh, Yi‐Ling Chen, Soo Weei Ng, David Cain, Rachel Etherington, Clare Hardman, Graham Ogg

**Affiliations:** ^1^ Medical Research Council Human Immunology Unit Radcliffe Department of Medicine Medical Research Council Weatherall Institute of Molecular Medicine University of Oxford Oxford UK; ^2^ NIHR Oxford Biomedical Research Centre Oxford University Hospitals Oxford United Kingdom

**Keywords:** Psoriasis, Acyloxyacyl hydrolase (AOAH), CD1a, Phospholipase A2, IL‐22

## Abstract

Psoriasis is a chronic inflammatory skin disease characterized by Th17 responses. Recent evidence has identified Langerhans cells to have a key role in disease pathogenesis, with constitutive high expression of CD1a and capacity to present lipid antigens to T cells. Phospholipase A_2_ enzymes generate neolipid antigens for recognition by CD1a‐reactive T cells; however, the broader enzymatic pathways of CD1a lipid ligand generation have not been thoroughly investigated. In this study, we used immunofluorescence of skin and ELISpot analyses of CD1a‐reactive T cells to investigate the role of the lipase acyloxyacyl hydrolase (AOAH) in CD1a ligand generation with relevance to the pathogenesis of psoriasis. We found that the PLA_2_ activity of rAOAH leads to the activation of circulating CD1a auto‐reactive T cells, leading to the production of IFN‐γ and IL‐22. Circulating AOAH‐responsive CD1a‐reactive T cells from patients with psoriasis showed elevated IL‐22 production. We observed that AOAH is highly expressed in psoriatic lesions compared to healthy skin. Overall, these data present a role for AOAH in generating antigens that activate circulating lipid‐specific CD1a‐restricted T cells and, thus, contribute to psoriatic inflammation. These findings suggest that inhibition of PLA_2_ activity of AOAH may have therapeutic potential for individuals with psoriasis.

## Graphical abstract

Acyloxyacyl hydrolase (AOAH), a lipase with PLA2 activity is highly expressed in lesional psoriatic skin. PLA2 activity of AOAH was found to activate CD1a‐reactive T cells. Individuals with psoriasis had elevated IL‐22 production by AOAH‐responsive circulating CD1a‐reactive T cells.

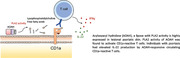

## Introduction

Psoriasis is a chronic immune‐mediated inflammatory skin condition that has a global prevalence of 2–3%. Psoriasis has several subphenotypes, however, *Psoriasis vulgaris* is the most prevalent manifestation. Psoriasis has a strong genetic basis with several linked loci [1], and immunopathology characterized by involvement of a cascade of immune cells including CD4^+^ and CD8^+^ T cells, mast cells, NKT cells, NK cells, macrophages, neutrophils, DCs, and innate lymphoid cells (ILCs) [1]. The dysregulation of the skin immune system is thought to promote hyperproliferation of keratinocytes, which results in the presence of thick scaly plaques. Psoriasis has long been known to be associated with a lesional T‐cell response [2] and subsequent studies characterized the role of IFN‐γ and IL‐12 [3, 4]. The discovery of increased numbers of IL‐17A‐producing T cells, in addition to the Th‐17 polarizing cytokine IL‐23, has demonstrated a central role for Th17‐responses in the pathogenesis of psoriasis [5, 6, 7, 8].

In addition to peptide antigens presented by MHC molecules, the family of MHC‐class‐1‐like nonpolymorphic CD1 molecules, encoded outside of the MHC cluster, has emerging roles in human disease. CD1 molecules CD1a, CD1b, CD1c, and CD1d can present foreign and self‐lipids for recognition by T cells [9, 10]. Each of these isoforms differs in size of antigen‐binding groves, intracellular trafficking patterns [11], tissue distribution [12], and lipid ligand repertoire [13, 14, 15, 16]. CD1a is constitutively expressed on Langerhans cells (LCs), which can initiate both innate and adaptive immune responses to skin‐relevant antigens. Furthermore, CD1a is expressed on thymocytes and some dermal DCs [17] and ILCs [18]. TNF‐α and IL‐1ß are cytokine cues that facilitate some LCs migration to local‐draining LNs; and LCs from patients with early‐onset psoriasis are stubborn to these cues, in support of their role in the pathogenesis of psoriasis [19]. In blood, CD1a‐autoreactive T cells are present that express skin‐homing receptors, such as cutaneous lymphocyte antigen, CCR4, and CCR10 [20]. CD1a‐autoreactive T cells are found in healthy skin [21] and were shown to be activated in a CD1a‐dependent manner in vitro displaying a predominant IL‐22‐ expressing phenotype [20]. IL‐22 forms a key component of skin homeostatic antibacterial immunity, where IL‐22 stimulation induces the production of ß‐defensin‐2 and increases keratinocyte proliferation, tissue‐remodeling, and wound healing [22, 23]. However, IL‐22‐expressing T cells have been implicated in the pathogenesis of psoriasis [24] and research has shown the role of lipid‐specific T‐cells responses to be an important factor in skin inflammatory conditions.

Fatty acids and lysophosphatidylcholine (LPC), identified as antigens for CD1a‐autoreactive T cells [25], can be generated by degradation of the ubiquitous membrane phospholipid, phosphatidylcholine by phospholipase A_2_ (PLA_2_) through hydrolysis of sn‐2 acyl bonds. Exogenous sources of PLA_2_, such as bee and wasp venom and house dust mite‐derived PLA_2_s have been shown to activate skin‐derived and circulating CD1a‐reactive T cells in a CD1a‐ and PLA_2_‐dependent manner [26, 27]. Increased PLA_2_ activity in lesional psoriatic skin was also demonstrated by increased levels of LPC, a known permissive CD1a ligand [28]. Endogenous PLA_2_s have also been described in lesional psoriasis skin such as the cytosolic‐PLA_2_δ [29]. Endogenous phospholipase (PLA2G4D), transferred to local CD1a‐expressing APCs in exosomes, triggered by IFN‐α, led to the generation of neolipid antigens and subsequent activation of CD1a‐restricted skin and circulating CD1a‐autoreactive T cells [30].

Acyloxyacyl hydrolase (AOAH) is a lipase that plays a critical role in the detoxification of LPS, a major constituent of the outer membrane of Gram‐negative bacteria [31, 32]. AOAH is typically found at low levels in circulating cells including neutrophils, monocytes, macrophages, immature dendritic cells, and NK cells, and it can be proteolytically processed into an endocytic and secreted form. [31, 33–35]. In addition to strong activity toward LPS, AOAH has also been shown to have PLA_2_ function [36]. Phospholipids acting as substrates for AOAH were confirmed with their crystal structure in complex with ligands including phosphatidylcholine [37]. In mouse studies, AOAH expression is seen to be upregulated in phagocytic cells in response to LPS [38], and downregulated in DCs by IL‐4 [33], but this has not been extensively studied in humans.

Psoriasis is characterized by the infiltration of immune cells in lesional skin, including monocytes, T cells [1], and abundant presence of neutrophils in lesions serves as histopathologic hallmarks of the condition [39]. Given the known expression of AOAH in neutrophils and other immune cells, we reasoned that AOAH may be upregulated in psoriatic lesions and sought to explore the role of AOAH and CD1a‐reactive T‐cell responses in the pathogenesis of psoriasis.

## Results

### AOAH activates circulating CD1a‐reactive T cells

**Figure 1:**
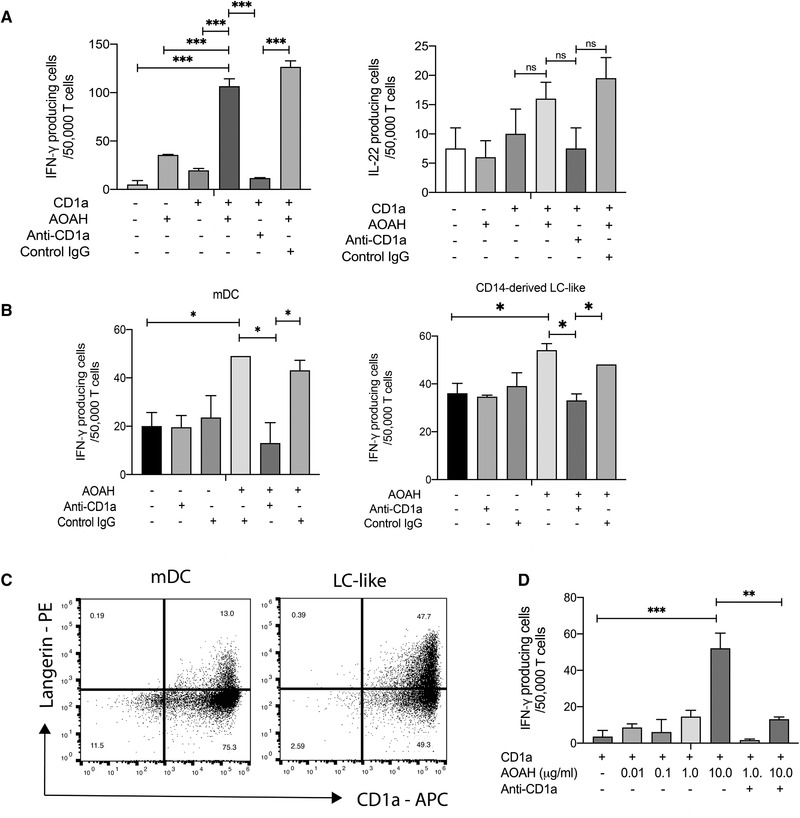
**AOAH generates CD1a ligands that activate circulating CD1a‐reactive T cells from healthy controls**. CD3^+^ T cells were isolated from healthy donors and incubated overnight with CD1a‐transfected K562 (CD1a‐K562) or mock transfected K562 cells (EV‐K562) pulsed with recombinant‐AOAH (rAOAH) (A‐D). The ratio of K562s, mDCs, or LC‐like cells to T cells was 1:2, with 25 000 APCs and 50 000 CD3^+^ T cells. IFN‐γ (left) and IL‐22 (right) expression was measured by enzyme‐linked immunospot (ELISpot) in the absence or presence of anti‐CD1a antibody and rAOAH from healthy volunteers (A). CD14^+^ cells isolated from healthy volunteers were differentiated into DCs and Langerhans‐like cells (LC‐like) in vitro and pulsed with rAOAH overnight and cocultured with autologous T cells. T‐cell responses were determined by IFN‐γ production in ELISpots (B). Langerin and CD1a expression on mDCs and LC‐like cells was determined by flow cytometry (C). To determine the dose‐dependent effect of AOAH, increasing 10‐fold concentrations of rAOAH, up to 10 μg/mL were used to pulse K562s (D). Data are representative of at least three donors from three independent experiments. Bars represent SE of mean and were analyzed using one‐way ANOVA *p < 0.033; **p < 0.002; ***p < 0.0001.

To determine whether AOAH enzyme activity could generate neolipid antigens recognized by CD1a‐restricted T cells, polyclonal CD3^+^ T cells isolated from healthy volunteers were coincubated with CD1a‐transfected K562s (CD1a‐K562) or empty vector‐transfected K562 (EV‐K562), pulsed overnight with recombinant AOAH (rAOAH). AOAH is an LPS detoxifying enzyme, therefore, enzymatic activity toward LPS deacylation was assessed in vitro. LPS deacylation was performed under pH‐controlled buffer conditions, namely at pH 5 and 7.5 for 1 h at 37 °C. Deacylated LPS ability to stimulate the production of IL‐6 in PMA differentiated Thp‐1 cells, was significantly diminished with increasing rAOAH concentrations (Supporting information Figure S1). K562 cells are HLA‐low and, therefore, minimize confounding alloreactivity, serving as comparator APCs to support the testing of CD1a‐reactive responses across populations [21, 27, 30, 40]. We noted a significant increase in the IFN‐γ T‐cell response upon coculture with AOAH‐treated CD1a‐K562, compared to T cells coincubated with unpulsed CD1a‐K562s, or rAOAH‐pulsed EV‐K562s (Figure 1A). There was a slight increase in response by T cells cocultured with unpulsed CD1a‐K562, representing T‐cell responses to endogenous K562s lipids presented on transfected CD1a, as has been observed previously [21]. The CD1a‐reactive T‐cell response seen with rAOAH was inhibited by anti‐CD1a antibody, however, not by its isotype control, confirming a CD1a‐mediated T‐cell response. Interestingly, circulating CD3^+^ polyclonal T cells from healthy volunteers did not produce significant levels of IL‐22 when cocultured with K562s pulsed with rAOAH, but this may have reflected the use of fetal calf serum rather than human serum which can amplify such responses (Figure 1A). We further examined AOAH‐responsive CD1a‐reactive T‐cell responses using autologous monocyte‐derived DCs (mDCs) and monocyte‐derived LC‐like cells as CD1a‐expressing APCs. In vitro derived mDC or LC‐like cells were pulsed with rAOAH overnight and incubated with autologous peripheral blood T cells. CD1a‐reactive T‐cell responses were detected within polyclonal T cells from healthy individuals, which were inhibited by anti‐CD1a antibody (Figure 1B). For mDCs and LC‐like cells, peptide‐MHC‐TCR and CD1c‐TCR interactions were blocked with HLA‐DR, HLA‐A, B, C, and CD1c blocking antibodies. CD1a expression was greatly increased in mDCs; and Langerin and CD1a, were both induced in LC‐like cells upon in vitro differentiation (Figure 1C). The gating strategy for the analysis of mDCs and LC‐like cells is shown in Supporting information Figure S2. The reactivity of polyclonal T cells from healthy individuals was amplified showing a 10‐fold increase in response up to 10 μg/mL rAOAH, confirming a dose dependence (Figure 1D). Overall these data demonstrated that AOAH generates stimulatory antigens that activate circulating CD1a‐restricted polyclonal T cells.

### AOAH activates CD1a‐autoreactive T cells from individuals with psoriasis to produce IFN‐γ and IL‐22

**Figure 2:**
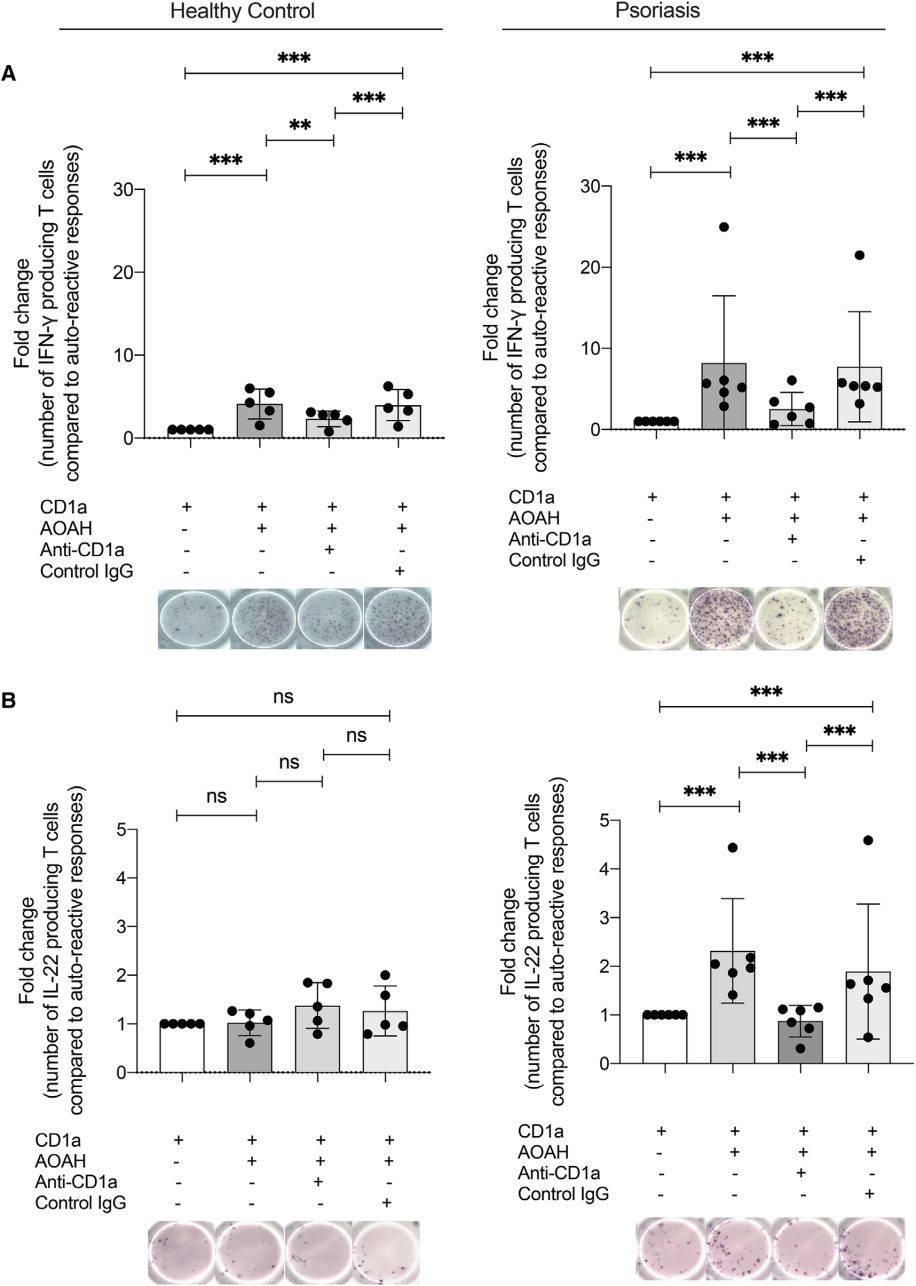
**CD1a‐reactive cells from psoriasis patients produce IL‐22 in the presence of AOAH**. AOAH activated circulating CD1a‐reactive T cells from healthy volunteers and psoriasis patients to express IFN‐γ and IL‐22. CD3^+^ T cells were isolated from healthy donors (n = 5 for IFN‐γ and n = 5 for IL‐22) and psoriasis patients (n = 6 for IFN‐γ and n = 6 for IL‐22) and incubated overnight with CD1a‐K562 or EV‐K562 cells pulsed with rAOAH. T cells (50 000) were cocultured with 25 000 K562s overnight and IFN‐γ and IL‐22 were measured by ELISpot in the presence or absence of anti‐CD1a antibody or IgG isotype. Data are represented as a fold change relative to CD1a‐autoreactive responses. The ELISpot data for each group of donors are cumulative three independent experiments. ELISpot images beneath graphs are representative of duplicates for each condition. Bars represent SE of mean and were analyzed using two‐way ANOVA with Tukey's multiple comparison *p < 0.033; **p < 0.002; ***p < 0.0001.

Next, we examined IL‐22 and IFN‐γ responses to AOAH in a larger cohort of healthy adults and individuals with psoriasis. CD1a‐dependent T‐cell responses to rAOAH were assessed by ELISpot assays using CD1a‐K562 as target cells to minimize APC variability. Activation of circulating CD1a‐reactive polyclonal T cells from both healthy volunteers and patients with psoriasis was consistent (Figure 2), when cocultured with CD1a‐K562s pulsed overnight with rAOAH. Increases in IFN‐γ production were significant in both healthy and patient cohorts (Figure 2A). Interestingly, IL‐22 production was not significantly increased by T cells from healthy volunteers (Figure 2B, left), whereas circulating polyclonal T cells from patients with psoriasis showed a significant increase in production of IL‐22 in a CD1a‐dependent manner in response to rAOAH (Figure 2B, right). These responses were blocked with anti‐CD1a antibody, with isotype controls showing no effect. The combined data highlight a role of AOAH in driving CD1a‐dependent T cells to produce IFN‐γ and IL‐22.

### CD1a‐autoreactive IFN‐γ and IL‐22 producing T‐cell clones activated by AOAH

**Figure 3:**
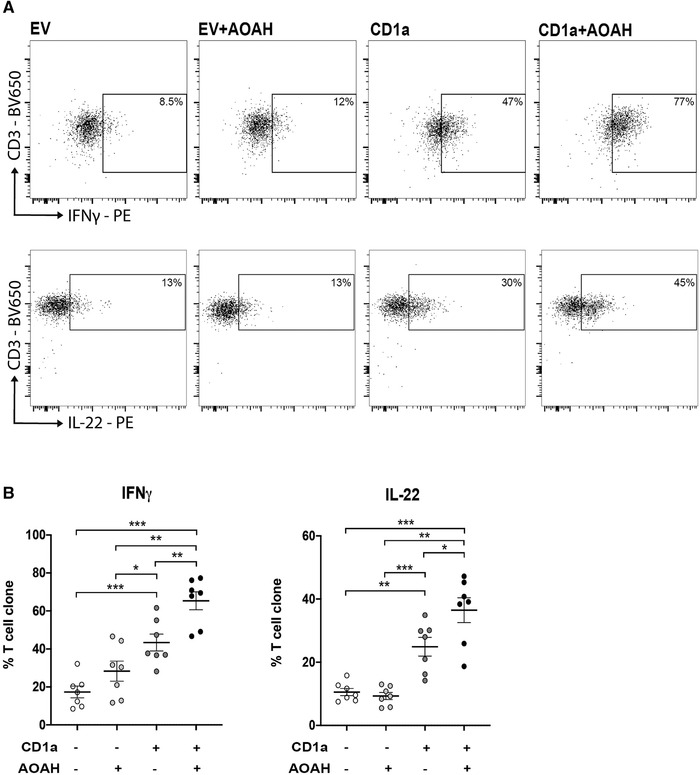
**AOAH increases IFN‐γ and IL‐22 production by CD1a‐autoreactive T‐cell clones**. IFN‐γ and IL‐22 producing CD1a‐reactive T‐cell clones were individually cocultured with EV‐K562 or CD1a‐K562 cells for 4 h with or without rAOAH pretreatment overnight. IFN‐γ and IL‐22 production were assessed by flow cytometry, with images representative of one clone for IFN‐γ and one for IL‐22 (A). A panel of seven CD1a‐autoreactive IFN‐γ and IL‐22 T‐cell clones showing percentage of AOAH responsive T‐cell clones determined by flow cytometry (n = 7) (B). The data are representative of three independent experiments with error bars representing SE of mean and were analyzed using two‐way ANOVA with Tukey's multiple comparison *p < 0.033; **p < 0.002; ***p < 0.0001.

Autoreactive T cells in humans that recognize endogenous lipid antigens presented on CD1a^‐^ are recognized as a normal component of the adaptive immune system [21]. To further confirm T‐cell functionality, we established CD1a‐reactive T‐cell clones and expanded to sufficient numbers to test whether AOAH could induce IFN‐γ and IL‐22 in a CD1a‐specific manner. CD1a‐transfected K562s were pulsed with rAOAH overnight and cocultured with IFN‐γ and IL‐22 producing CD1a‐autoreactive T‐cell clones for 4 h. Percentage of T‐cell clone activation was assessed by cytokine production with a FACS‐based secretion assay. We found that cytokine production in the T‐cell clones cultured with unpulsed CD1a‐K562s increased, representative of confirming their CD1a‐autoreactivity (Figure 3A). Notably, the cytokine production was significantly increased in T‐cell clones cocultured with CD1a‐K562s pulsed with rAOAH (Figure 3A), which was broadly observed in a cohort of CD1a‐autoreactive T‐cell clones (Figure 3B). We also observed a trend toward non‐CD1a‐specific responses with respect to IFN‐γ production generated by rAOAH, with EV‐K562s pulsed with rAOAH compared to the unpulsed control, however, this was not significant.

To ensure AOAH‐specific responses are not due to a contaminant protein in our rAOAH preparation and, hence, to confirm AOAH‐specificity, rAOAH was immunoprecipitated with anti‐human AOAH antibody, and CD1a‐reactive T‐cells responses assessed by IL‐22 producing CD1a‐restricted T‐cell clones. Removing AOAH protein attenuated AOAH‐specific IL‐22 production by CD1a autoreactive T‐cell clones, suggesting an AOAH‐specific CD1a‐reactive responses observed in our findings (Supporting information Figure S3). Collectively, these data fit with our previous studies showing elevated CD1a‐autoreactive T cells in psoriatic lesional skin [30] and further support the role of AOAH in generating stimulatory CD1a‐ligands that further activate CD1a‐autoreactive T cells to produce IFN‐γ and IL‐22.

### CD1a‐reactive responses are dependent on the PLA_2_ activity of AOAH

**Figure 4:**
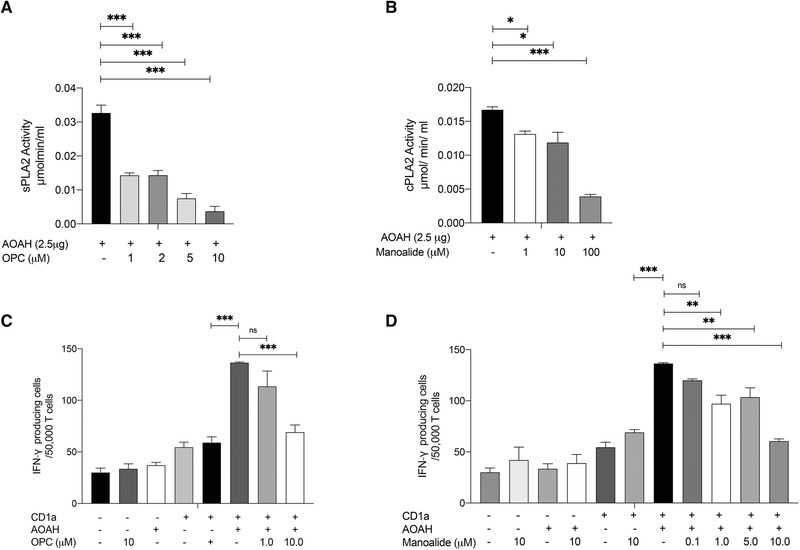
**The secreted‐PLA_2_ (sPLA_2_) and cytosolic‐PLA_2_ (cPLA_2_) activity of AOAH generates antigens for presentation by CD1a to T cells**. sPLA_2_ and cPLA2 activity of AOAH was detected by biochemical assays. Oleyloxyethyl phosphorylcholine (OPC) and manoalide, PLA_2_ inhibitors, we added at increasing concentrations to the PLA_2_ activity assays (A and B, respectively). Polyclonal T cells from healthy controls were cocultured with CD1a‐K562 or EV‐K562 cells pulsed with rAOAH in the absence and presence of increasing concentrations of OPC and Manoalide. CD1a‐autoreactive T‐cell responses were measured by IFN‐γ and IL‐22 ELISpots (C and D, respectively). Data representative of at least three donors from three independent experiments, with each condition done in duplicates. Bars represent SE of mean and were analyzed using one‐way ANOVA *p < 0.033; **p < 0.002; ***p < 0.0001.

AOAH has recently been found to associate with phosphatidylcholine and fatty acids, visualized with the crystal structure [37]. We next determined whether AOAH used in these experiments had PLA_2_ activity and whether this was responsible for generating stimulatory CD1a ligands that are known to activate CD1a‐restricted T cells including lysophospholipids and fatty acids [25]. Phospholipases can exist in a secreted and cytosolic form. Cytosolic‐PLA_2_ exhibits specificity toward arachidonic acid, however, other PLA_2_s, including sPLA_2_s, can hydrolyze different fatty acids at the sn‐2 position [41]. We measured the sPLA_2_ and cPLA_2_ activity of rAOAH in vitro. This was accomplished by colorimetric detection of thiol release from diheptanoyl thio‐PC substrate for sPLA_2_ and arachidonoyl thio‐PC for cPLA_2_. Interestingly, AOAH has both sPLA_2_ and cPLA_2_ activity in vitro and these activities were significantly inhibited by known PLA_2_ inhibitors manoalide and oleyloxyethyl phosphorylcholine (OPC), demonstrated with increasing inhibitor concentrations (Figure 4A and B). To determine whether the PLA_2_ activity of AOAH promoted CD1a reactivity, we pulsed K562‐EV and K562‐CD1a with AOAH in the absence or presence of increasing concentrations of manoalide and OPC. CD1a‐reactive responses as measured by IFN‐γ ELISpots were significantly inhibited in the presence of both PLA_2_ inhibitors (Figure 4C and D). There was no inhibition of unpulsed K562‐CD1a responses, suggesting lack of nonspecific effects as demonstrated previously [26]. Collectively, these data demonstrate that the PLA_2_ activity of AOAH has the capacity to generate neolipid antigens for recognition by CD1a‐reactive T cells.

### AOAH protein is detected in psoriatic lesional skin but not in healthy skin

**Figure 5:**
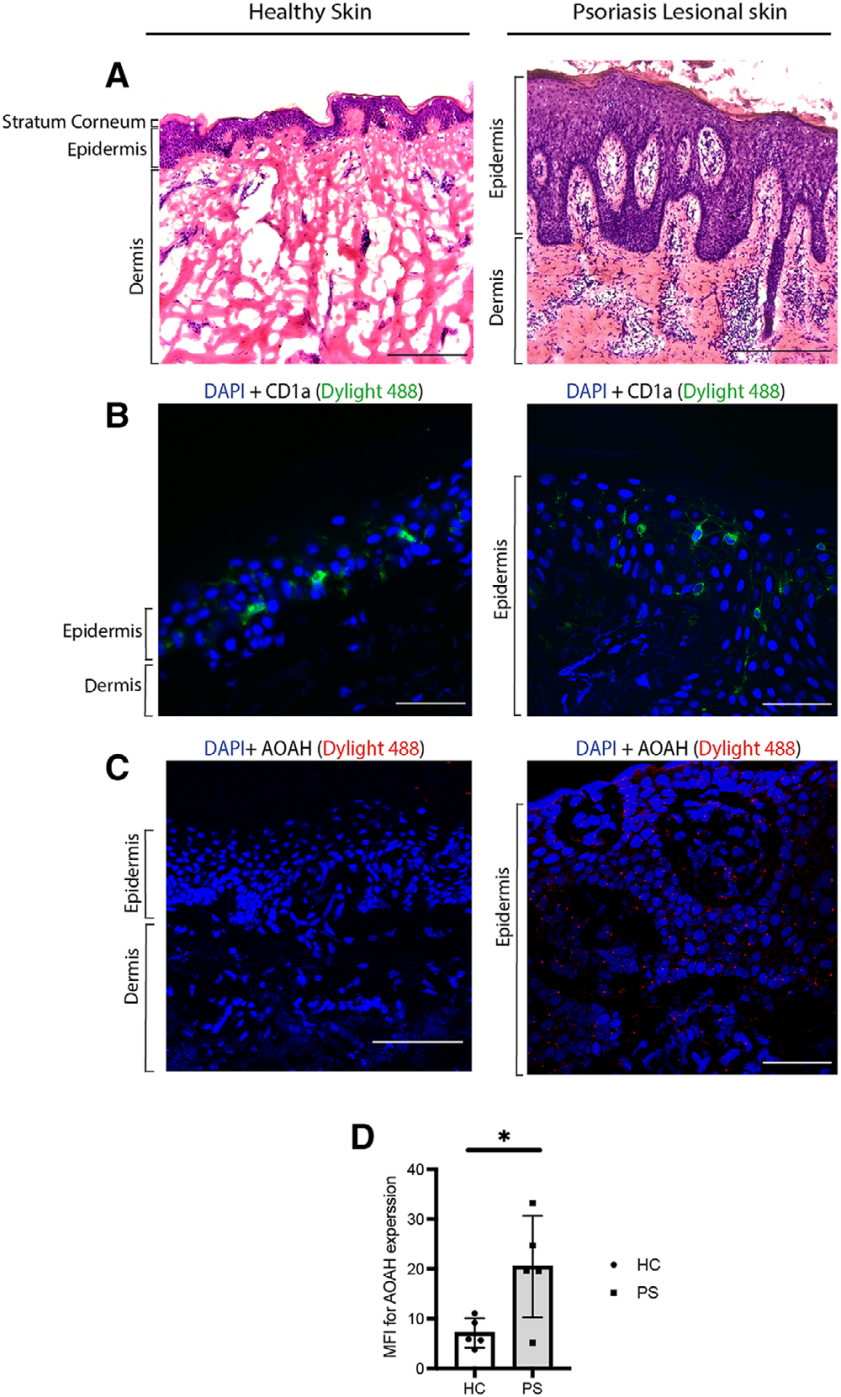
**AOAH protein detected in lesional psoriatic skin**. HE staining and immunofluorescence on cryosections of healthy and lesional skin with left panel showing healthy skin and the right representing psoriatic lesional skin, Bar 100 μm. Images were captured by bright field and confocal microscopy. H&E was performed on 7 μm cryosections of healthy skin and lesional skin (A). IF on healthy skin and lesional cryosections fixed in ice‐cold acetone (B, C). CD1a staining was performed with Dylight 488 (Green) conjugated secondary antibody for anti‐CD1a. AOAH staining with Dylight 594 (Red) conjugated secondary antibody on healthy and lesional skin (C). Tissues sections were mounted with mounting media containing DAPI. High AOAH in psoriatic skin compared to healthy skin was detected in five healthy and five patients with psoriasis (D).

The GEO profiles database was employed to explore publicly available RNA‐seq addressing skin inflammatory diseases. Nair and colleagues [42] measured total RNA from skin biopsies of healthy individuals, as well as uninvolved nonlesional skin and lesional skin from the same psoriatic individuals [42]. AOAH was differentially expressed in lesional and uninvolved nonlesional and healthy skin. AOAH showed increased expression in lesional skin, compared to both uninvolved skin from the same donors and individuals with no psoriasis (GEO dataset ID GSE13355, Supporting information Figure S4). To confirm whether AOAH was presented in lesional skin at the protein level, skin sections from healthy or psoriatic donors were collected. H&E staining of lesional skin revealed typical characteristics associated with psoriasis including psoriasiform hyperplasia, parakeratosis, and infiltration of immune cells (Figure 5A, left healthy, right psoriasis). Single‐color immunofluorescence (IF) for CD1a and AOAH was conducted on healthy skin cryosections. As expected, CD1a^+^ cells were detected in the epidermis, representing LCs (Figure 5B, left). However, AOAH was not detected in either the epidermis or the dermis of healthy skin, consistent with the RNA‐sequence data (Figure 5C, left). Next, we sought to determine AOAH and CD1a protein expression by IF in lesional psoriatic cryosections. IF staining with anti‐CD1a antibodies showed increased frequency of CD1a^+^ cells in psoriatic epidermis compared to healthy skin, consistent with a key role for these cells in lesional skin pathology (Figure 5B, right). Using IF with anti‐AOAH antibodies, we detected AOAH at significant levels in lesional skin (Figure 5C, right, and 5D). Morphological and architectural analyses suggested AOAH expression by different populations; therefore, we aimed to further characterize the relevant AOAH‐expressing cells.

### AOAH and CD1a coexpressing cells in psoriatic lesional skin

**Figure 6:**
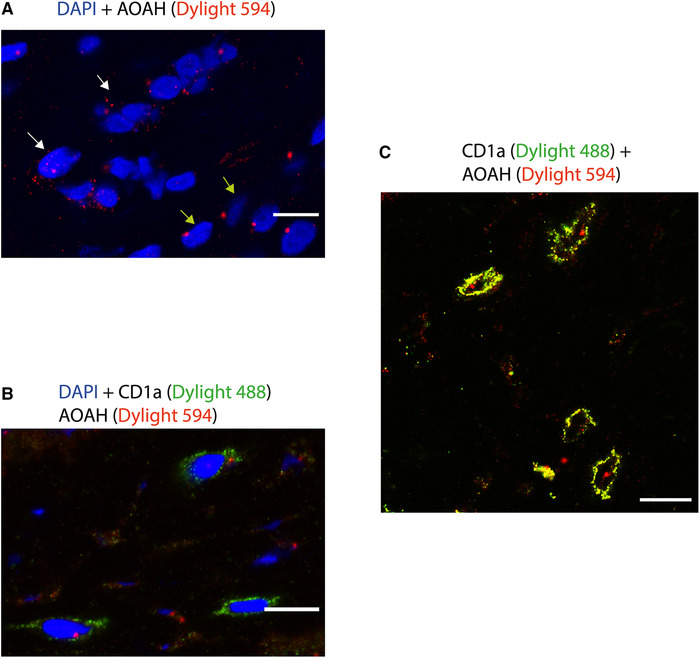
**AOAH expressed in immune cells with multilobed nuclei and coexpressed in cells positive for CD1a** immunofluorescence (IF) for psoriatic lesional skin cryosections for AOAH and CD1a showing different AOAH^+^ cells with multilobed nuclei, and lymphoid‐like, and CD1a^+^ AOAH^+^ coexpressing cells. IF was performed on 7 μm lesional psoriatic skin cryosections, with secondary antibodies for AOAH conjugated to Dylight 594 (Red) and secondaries antibody for CD1a conjugated to Dylight 488 (Green), BAR 10 μm. Images were captured by confocal microscopy. (A) AOAH^+^ neutrophil‐like multilobed nuclei cells in lesional epidermis (white arrows), in addition other mononuclear cells (green arrows) were also AOAH positive. (B and C) AOAH and CD1a coexpressing cells possibly representing LC population in psoriatic lesional skin. The results for single and dual staining for AOAH and CD1a were consistantly observed in at least three patients with psoriasis in three independent experiments.

AOAH is known to be expressed by some epithelia and other cells, such as macrophages, NK cells, neutrophils, and DCs ( [43]), and is localized to endosomes and lysosomes [44]. We sought to determine whether AOAH and CD1a are coexpressed in APCs, which may suggest an endogenous role for AOAH lipase activity in generating neolipid antigens. Dual staining was performed on lesional cryosections for AOAH and CD1a. AOAH positive cells were detected, many of which had multilobed nuclei features compatible with neutrophils (Figure 6A), and in other cells which had a more lymphoid morphology. Furthermore, neutrophils are known to produce AOAH, which may be secreted locally, retaining its enzymatic activity [44]. We also detected AOAH and CD1a coexpressing cells (Figure 6B and C). CD1a and AOAH dual‐positive cells may represent LCs or other CD1a‐expressing mononuclear phagocytic cell populations. We found that neutrophils constitutively express AOAH at the protein level and TNF‐α was able to induce AOAH secretion (Supporting information Figure S5). These findings further suggest an important role for both secreted and cytosolic forms of AOAH in psoriatic inflammation.

## Discussion

Evidence of T cells having a prominent role in the pathogenesis of psoriasis is now well established, and therapeutics targeting key T‐cell pathway inflammatory mediators have shown to be highly effective [45]. In addition to TNF‐α, IL‐23, IL‐12, and IFN‐γ as key inflammatory mediators in disease pathology, IL‐22 is now increasingly recognized as a notable component of the inflammatory cascade. Expression of IL‐22 in lesional psoriatic skin is higher when compared to nonlesional and healthy skin and the IL‐22 receptor is predominantly expressed on epithelial cells including keratinocytes in the skin [46]. IL‐22 has an important role in host defense and antimicrobial protection, however, when overexpressed it contributes to keratinocyte parakeratosis, acanthosis, and papillomatosis, which are hallmarks of psoriasis [23, 46].

CD1a is constitutively expressed at high levels on LCs which has altered function and migratory patterns in psoriasis [47, 48]. Upon activation by permissive CD1a‐presented ligands, CD1a‐autoreactive T cells can express IFN‐γ and IL‐22, among other effector cytokines [21]. Phenotypic properties of these cells suggest that IL‐22 production by CD1a‐autoreactive T cells is a prominent effector function of the CD1a‐autoreactive T‐cell repertoire. IL‐22 producing T helper cells have also been characterized to express skin homing chemokine receptors and these subsets of cells show the greatest and most frequent CD1a‐responses and have an essential role in skin immunity [21, 40, 49].

CD1a‐autoreactive T cells are elevated in the blood and skin of patients with psoriasis [30] and lipid antigens for CD1a‐autoreactive T cells [25], can be generated by degradation of the ubiquitous membrane phospholipid, PLA_2_ through hydrolysis of sn‐2 acyl bond [30]. We found AOAH was highly expressed at the protein level in lesional psoriatic skin compared to healthy controls. AOAH has been shown to have PLA_2_ function, which was confirmed in this study and by its crystal structure in complex with phosphatidylcholine, showing phospholipids as substrates for this enzyme [37]. In the present study, we showed that PLA_2_ activity of AOAH activated circulating CD1a‐autoreactive T cells to produce IFN‐γ in patient and control samples. However, IL‐22 production was significantly higher in T cells derived from patients with psoriasis. Endogenous and exogenous PLA_2_ enzymes have been shown to generate neolipid antigens for recognition by CD1a‐reactive T cells in skin disease [26, 30, 50]. The findings in this study further support the role of nonpeptide antigens in psoriasis as key activators of T cells, and PLA_2_ activity of AOAH as a significant contributing factor to the disease pathogenesis. Furthermore, IFN‐γ and IL‐22 CD1a‐autoreactive T‐cell clones also showed increased activation when cocultured with CD1a‐K562s pulsed with rAOAH, compared with CD1a‐autoreactive responses.

AOAH has been described to be differentially proteolytically processed into both a secreted and cytosolic form in phagocytic cells ([43, 44]). Here, we showed that AOAH exhibited both sPLA_2_ and cPLA_2_ activity in vitro, which was inhibited by irreversible PLA_2_ inhibitors manoalide and OPC. Cytosolic PLA_2_s (cPLA_2_) show a preference for specific phospholipids at the sn‐2 position, whereas other PLA_2_s (sPLA_2_) hydrolyze can show broad substrate preference [51]. Furthermore, we were able to show that the PLA_2_ activity of AOAH was primarily responsible for activating circulating CD1a‐autoreactive T cells. Part of the AOAH structure is a saposin domain; saposin proteins participate in membrane and lipid binding, which may assist AOAH to access membrane phospholipids [37, 52].

Psoriasis is characterized by inflammatory infiltrate of leukocytes and neutrophil accumulation within the dermis and epidermis is a hallmark feature of psoriasis [53, 54]. Expression of AOAH is primarily attributed to phagocytic cells such as neutrophils, macrophages, DC, and also NK cells [55]. Neutrophils are found in lesional psoriatic skin in abundance [39] and were found to have constitutive AOAH protein expression with secretion enhanced by inflammatory mediators such as TNF‐α. Therefore, neutrophils can serve as an important source of AOAH in lesional psoriatic skin. Interestingly, in addition to single AOAH positive cells, we also detected cells coexpressing CD1a and AOAH in psoriatic lesions. This may suggest AOAH could potentially generate ligands for CD1a in an autocrine manner. CD1a lacks the tyrosine‐based cytosolic sorting motif, therefore, it localizes in the early endocytic compartments, where the loading of lipid antigens can take place. [56]. AOAH and CD1a coexpressing cells in lesional skin also raise the possibility of AOAH generating CD1a‐ligands internally, however, this requires further investigation. From our findings, AOAH protein was not detected in skin from healthy volunteers, suggesting a key role for this enzyme in disease pathology.

Here, we provide evidence for AOAH PLA_2_ activity, in generating neolipid antigens that activate circulating CD1a‐autoreactive T cells. CD1a‐autoreactive T cells from psoriasis patients showed elevated IL‐22 expression in the presence of AOAH, which was enriched in psoriatic lesions. These findings also have therapeutic potential. In addition to therapies inhibiting T cell and innate cell‐derived cytokines, such as IL‐17A, TNF‐α, and IL‐23/12, these data support the development of parallel approaches to inhibit the PLA_2_ activity of AOAH.

## Materials and methods

### Cell lines

EV‐K562 and CD1a‐transfected K562 (CD1a‐K562) cells (a gift from B. Moody, Brigham and Women's Hospital, Harvard Medical School, Boston, MA) were maintained in RPMI 1640 medium supplemented with 10% FCS, 100 IU/mL penicillin, 100 μg/mL streptomycin (Sigma‐Aldrich), 2mM l‐glutamine (Gibco), 1 × nonessential amino acids (NEAAs) (Gibco), 1 mM sodium pyruvate (Gibco), 10 mM HEPES (Gibco), 500 μM 2‐ME (Gibco), and 50 μg/mL G418 antibiotic (Thermo Fisher Scientific).

### Isolation and culture of human T cells

Healthy adult donors and Psoriasis patients with mild‐to‐moderate and moderate‐to‐severe form of psoriasis with PASI scores between 5 and 26 were recruited under local ethics approval Local ethical approval was given by the Oxford Ethics Committee (09/H0606/71). PBMC were isolated from healthy donors and psoriasis patients by Lymphoprep medium (Stem Cell Technologies) and T cells were further purified by magnetic‐activated cell‐sorting by CD3^+^ magnetic beads (Miltenyi Biotec) following the manufacturer's instructions, and maintained in RPMI 1640 medium (Sigma) supplemented with 10% FCS (Sigma), 100 IU/mL penicillin (Sigma), 100 μg/mL streptomycin, 2 mM l‐glutamine (Sigma), 1 × NEAA, 10 mM HEPES, 500 μM 2‐ME. T cells were maintained in culture for 2–3 days with IL‐2 (200 U/mL, BioLegend) before being used in experiments.

### Isolation and stimulation of neutrophils

Neutrophils were isolated from 8 mL of human anticoagulated blood by negative selection using the MACSxpress Whole Blood Neutrophil Isolation Kit (Miltenyi) according to the manufacturer's instruction. Isolated cells were stained for CD14 (BioLegend, HCD14), to select CD14 negative cells and CD15 (BioLegend, W6D3) and CD16 (BioLegend, 3G8) to confirm purity. Cells were maintained in RPMI 1640 medium (Sigma) supplemented with 10% FCS (Sigma), 100 IU/mL penicillin (Sigma), 100 μg/mL streptomycin, 2 mM l‐glutamine (Sigma), 1 × NEAA, 10 mM HEPES, 500 μM 2‐ME. For stimulation, 1 million cells were seeded in a 48‐well plate and stimulated with 50 ng/mL TNF‐α for 18 h. Supernatants were collected and stored at −80°C until analyzed by ELISA.

### AOAH ELISA

To detect AOAH in supernatants from neutrophils, Human Acyloxyacyl Hydrolase ELISA Kit (Abbexa, abx150541) was used according to manufacturer's instruction.

### CD14^+^ isolation and generation of human APCs

Autologous monocyte‐derived DCs and monocyte‐derived LC‐like cells were generated from CD14^+^ monocytes isolated from PBMCs. CD14^+^ cells were isolated from PBMCs by MACS, using CD14^+^ magnetic beads (Miltenyi Biotec) following the manufacturer's instructions. The cells were cultured in R10 (RPMI 1640 supplemented with 10% FCS, 100 IU/mL penicillin, 100 μg/mL streptomycin, 2 mM l‐glutamine, 1 × NEAA, 10 mM Hepes, 500 μM 2‐ME) at 37°C and 5% CO_2_. For mDCs, the culture media was constituted with GM‐CSF (100 ng/mL, BioLegend) and IL‐4 (10 ng/mL, BioLegend) for 5 days, on day 3, cells were replenished with differentiating cytokines at the same concentrations. CD1a (BioLegend, HI149) expression was confirmed by flow cytometry [57] and differentiated mDCs were used in elispots. For LC‐like cells, CD14^+^ monocytes were maintained in R10 for 3 days with 100 ng/mL GM‐CSF (BioLegend), 10 ng/mL IL‐4 (BioLegend), TGF‐ß (BioLegend) 10 ng/mL, TNF‐α (BioLegend) 10 ng/mL and, on day 3, the cells were replenished with differentiation cytokines at the same concentrations without IL‐4. CD1a (BioLegend, HI149) and Langerin (BioLegend, 10E2) expression was measured by flow cytometry and the cells were used in ELISpot assay.

### Activation assay of human CD1a‐autoreactive T‐cell clones

EV‐K562 and CD1a‐K562 cells were pulsed with 10 μg recombinant‐AOAH overnight at 37°C and 5% CO_2_. Following the overnight culture, K562 cells were washed to remove excess AOAH. K562 (2 × 10^5^) cells were cocultured with 1–5 × 10^5^ CD1a‐autoreactive T‐cell clones for 4 h. IFN‐Ɣ‐producing T‐cell clone culture was supplied with IL‐12 (1 ng/mL, BioLegend), IL‐18 (1 ng/mL, BioLegend), and IL‐2 (25 U/mL, BioLegend); and IL‐22‐producing T‐cell clone culture were supplied with IL‐6 (5 ng/mL, BioLegend), TNF‐α (5 ng/mL, BioLegend), and IL‐2 (25 U/mL, BioLegend). Activation of T‐cell clones was accessed by cytokine production of T cells using Secretion Assay (Miltenyi Biotec) following the manufacturer's instructions. Data were collected using a LSR Fortessa (BD Biosciences) and analyzed using FlowJo software (Tree Star Inc.).

### Skin biopsies and generation of frozen tissue blocks

Skin biopsies were acquired from psoriasis patients with moderate‐to‐severe psoriasis and healthy controls at the Dermatology Department, Churchill Hospital, Oxford under ethical approval. The biopsy site was typically right lower back from patients with PASI scores between 5 and 26. Biopsies were snap‐frozen and mounted in OCT compound for IF and HE experiments. OCT compound is water soluble and composed of glycols and resins for cryostat sectioning at −10°C and lower. OCT mounted tissues were stored at −80°C until required. Cryosections (7 μm) of mounted lesional and healthy skin biopsies were generated by Zoe Askham (Dermatology Department, Churchill hospital) onto Super Frost Plus slide (Fisher Scientific). The slides were fixed for 10 min in ice‐cold acetone and stored at −80°C.

### Secreted and cytosolic PLA_2_ biochemical activity assays

rAOAH was generated by Oxford Expression Technologies, using the *flash*BAC system. PLA_2_ activity of recombinant‐AOAH was detected using cPLA_2_ and sPLA_2_ kits (Caymans Chemicals) according to the manufacturer's protocol. For cPLA2, arachidonoyl thio‐PC substrate has AA at the *sn‐2* position of the glycerophospholipid. A free thiol is released by hydrolysis of thioester bond at *sn‐2* position by an enzyme with PLA2 activity, which is subsequently detected by DTNB ((5,5′‐dithiobis [2‐nitrobenzoic acid]). Of the PLA_2_ enzymes, cPLA_2_ exhibit specificity toward AA, whereas other PLA_2_s can hydrolyze any fatty acid at the *sn‐*2 position. For sPLA_2_ diheptanoyl phosphatidylcholine serves as a substrate instead. The free thiols released upon hydrolysis of *sn‐2* bond are detected by DTNB. For AOAH inhibitor studies, PLA_2_ inhibitors manoalide (ChemCruz, 75088‐80‐1) and OPC (Enzo, BML‐ EI118‐0010), which were added to AOAH at indicated concentrations. The inhibitors were dissolved in DMSO as described in the manufacture's data sheet and, therefore, DMSO was used as a control.

### Hematoxylin and eosin staining

Frozen 7 μm cryosections from healthy and psoriatic lesional skin tissue, stored at −80°C, were thawed at room temperature for 10–20 min and hydrated in PBS for 5 min. They were stained with H&E(Vector Laboratories, H‐3502) according to the manufacturer's instruction. Briefly, thawed and hydrated sections were completed immersed in Hematoxylin for 5 min followed by two changes rinse in distilled water. Bluing reagent was applied for 10–15 seconds, again followed by two changes rinse in distilled water. The slides were dipped in 100% ethanol for 10 s and incubated for 3 min in Eosin Y solution, followed by a rinse and dehydration in 100% ethanol. The slides were mounted with a xylene‐based mounting medium (Micromount, Leica Biosystems, 3801731).

### Immunofluorescence and confocal microscopy

Cryosections stored at −80°C were thawed at room temperature for 10–20 min and rehydrated in PBS for 10 min. The sections were then blocked in 2.5% horse serum (Vector Laboratories) for 1 h at room temperature to reduce nonspecific antibody binding. Slides were then placed in a dark damp chamber for incubation with primary and secondary antibodies. Primary antibodies for confocal microscopy were, CD1a (Mouse anti‐human, 1:100, BioLegend, HI149) and AOAH (Rabbit anti‐human, 1:100, ThermoFisher, PA5‐54490). Cryosections were incubated with primary antibodies prepared in TBS with 0.1% Tween‐20 (TBS‐T), for 2 h at room temperature. Secondary antibody staining for AOAH was done with VectaFluor Excel Amplified anti‐Rabbit IgG, Dylight 594 antibody kit (Vector Laboratories, DK‐1594) and for CD1a with VectaFluor anti‐Mouse IgG, Dylight 488 antibody kit (Vector Laboratories, DK‐2488) according to the manufacturer's instruction. Briefly, after primary antibody incubation, slides were washed in wash buffer provided in the kit for 5 min, followed by a 15 min incubation with amplifier antibody. Slides were once again washed for 5 min and incubated with Dylight488 or 594 VectaFluor reagent for 30 min. All steps were performed at room temperature and in a dark moist chamber. The slides were subjected to two further 5 min washes before being mounted in Vectashield, antifade mounting media with DAPI (Vector Laboratories, H‐1200‐10). For neutrophils, isolated cells were fixed onto slides with ice‐cold acetone for 10 min and washed 3× in TBS‐T 0.1% Tween‐20. After blocking in goat serum (ThermoFisher Scientific) for 20 min, the slides were washed in TBS‐T 0.1% Tween‐20 3× for 5 min. Primary antibody Rabbit anti‐human AOAH (ThermoFisher Scientific, PA5‐54490) as added onto cells at 1:100 in TBS‐T 0.1% Tween‐20 and incubates at 4°C overnight in a moist chamber. Secondary antibody Goat anti‐Rabbit IgG conjugated to Alexa Fluor 568 was used at 1:1000 concertation for 1 h at room temperature. Images were acquired on ZEISS 880 inverted confocal microscope and images were processed by ZEN imaging software (Black edition, 2.1 SP3 LSM). To avoid pixel saturation, laser intensity and amplifier gains were adjusted accordingly.

### ELISpot experiments

On day 1, ELISpot plates (Merck Millipore Corp.) were pretreated with 35% ethanol for less than a minute, washed with water five times, and coated with 15 μg/mL anti‐IFN‐γ or 10 μg/mL anti‐IL‐22 (Mabtech) overnight at 4°C. K562 cells, mDCs, or LC‐like cells were washed in RPMI 1640 supplemented with 10 mM HEPES (R‐HEPES) and resuspended in RPMI 1640 medium supplemented with 10% fetal calf serum, 100 IU/mL penicillin, 100 μg/mL streptomycin, and 2 mM l‐glutamine (R10). The cells were then pulsed with 10 μg recombinant‐AOAH overnight at 37°C and 5% CO_2_ at a density of 4 00 000 cells per 200 μL of media. Polyclonal CD3^+^ T cells isolated from PBMC by MACS separation were maintained in complete RPMI (see Isolation of human T cells). On day 1 of ELISpot, CD3^+^ T cells were rested overnight after washing twice in R‐HEPES, resuspended in R10 and incubated at 37°C and 5% CO_2_. On day 2, the ELISpot plates were washed five times in R‐HEPES and blocked for 30–60 min with R10. Following the blocking step, the plates were washed twice with RPMI 1640 medium. 50 000 T cells were cocultured with 25 000 K562 cells, mDCs, or LC‐like cells in each well, which were set‐up in duplicates. T cells alone were used as a negative control and T cells with 100 ng/mL PMA and 100 ng/mL ionomycin served as a positive control. CD1a‐transfected or empty vector‐transfected K562 cells were incubated with 10 μg/mL anti‐CD1a blocking antibody (Biolegend 300102, clone HI149) or 10 μg/mL IgG, isotype control (Biolegend 400153, clone MOPC‐21) for 1 h. For experiments with mDCs and LC‐like cells, 10 μg/mL anti‐HLA‐ABC (Biolegend 311423, clone W6/32) and 10 μg/mL anti–HLA‐DR blocking antibodies (Biolegend 307612, clone L234) and 10 μg/mL their IgG isotype controls (BD 555571, clone G155‐178) were added for 2 h before addition of T cells. CD1a blocking antibody (Biolegend 300102, clone HI149) or 10 μg/mL IgG, isotype control (Biolegend 400153, clone MOPC‐21) was also added for mDCs and LC‐like cells. APCs, K562s, or mDCs/LC‐like cells were coincubated with T cells overnight at 37°C and 5% CO_2_. On day 3, the plates were washed five times with R‐HEPES and incubated in the detection antibody for anti‐IFN‐γ or IL‐22 conjugated to biotin prepared at 1 μg/mL in PBS containing 0.05% FCS for 2 h at room temperature. The plates were washed in R‐HEPES five times and incubated with streptavidin‐alkaline phosphatase solution for 1 h at room temperature and the spots developed with an alkaline phosphatase conjugate kit (Bio‐Rad Laboratories). The spots were visualized and analyzed with the aid of an ELISpot plate reader (ELISpot Reader Classic; Autimmun Diagnostika gmbh). AOAH‐specific responses typically produce between 200–400 spots for IFN‐γ and 20–50 spots for IL‐22.

### AOAH IP

rAOAH protein was incubated with anti‐AOAH antibody (10‐50 μg, abx128358, Abbexa) coated Protein G magnetic beads for 2 h at 4°C. Beads were then separated by magnetic separation rack. Clear supernatant was collected for pulsing K562. The magnetic beads were washed and resuspended in SDS gel loading buffer and boiled at 95°C for 2 min. Samples were separated on a 4 to 12%, Bis‐Tris gel (NuPAGE, Invitrogen) and stained by SimplyBlue SafeStain (Invitrogen) following manufacturer's instructions.

### Enzymatic activity of rAOAH toward LPS

Deacylation of LPS was assayed at increasing concentrations of rAOAH (1, 2, 5, and 10 μg) toward 10 ng LPS in buffer (100 mM NaCl, 20 mM Tris‐HCl (pH 7.5) or sodium acetate (pH 5) at 37°C for 60 min. Reaction mixture was subsequently added to differentiated Thp‐1 cells to assess IL‐6 production according to manufacturer's instruction (Thermo Fisher).

### Statistics

All values are shown as means. Error bars represent SEMs. One‐way and Two‐way ANOVA (with multiple comparisons) were performed with Tukey's multiple comparison test using GraphPad Prism version 7.00 (GraphPad Software) to assess statistical significance: *p < 0.05; **p < 0.01; ***p < 0.005; and ****p < 0.0001.

## Conflict of interest

Competing interests: G. Ogg has served on advisory boards or holds consultancies or equity with Eli Lilly, Novartis, Janssen, Orbit Discovery, T‐Cypher Bio, and UCB Pharma, and has undertaken clinical trials with Atopix, Regeneron/Sanofi, Novartis, Roche, AnaptysBio. The authors have no other conflict of interest to declare.

### Peer review

The peer review history for this article is available at https://publons.com/publon/10.1002/eji.202149485.

**Abbreviations**
AOAH: acyloxyacyl hydrolase
CD1a‐K562: CD1a‐transfected K562s
cPLA_2_
: cytosolic PLA_2_s
EV‐K562: empty vector‐transfected K562
IF: immunofluorescence
ILCs: innate lymphoid cells
LCs: Langerhans cells
LPC: lysophosphatidylcholine
MACS: magnetic‐activated cell‐sorting
mDCs: monocyte‐derived dendritic cells
NEAAs: nonessential amino acids
OPC: oleyloxyethyl phosphorylcholine
rAOAH: recombinant AOAH
TBS‐T: TBS with 0.1% Tween‐20

## Supporting information



Supporting information

## Data Availability

The data that support the findings of this study are available in the manuscript and/or from the corresponding author upon request.
