# Supplementary material for: Phospholipase activity of acyloxyacyl hydrolase induces IL‐22‐producing CD1a‐autoreactive T cells in individuals with psoriasis
Source: Eur J Immunol. 2022 Jan 10;52(3):511–24. doi: 10.1002/eji.202149485 (PMC9302981; doi:10.1002/eji.202149485)
Supplement: Supplementary file 1 — Supporting information [file EJI-52-511-s001.pdf]

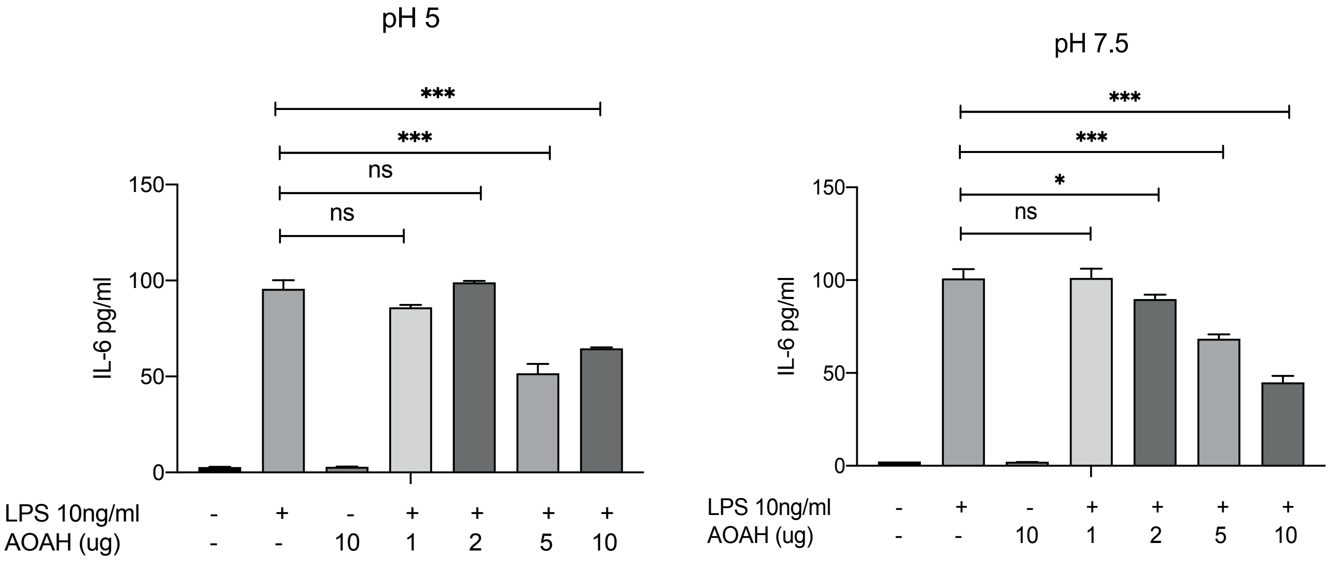

Figure S1

LPS (Sigma E. coli 0111.B4) deacylation by recombinant AOAH lessened IL-6 production in differentiated Thp-1 cells. Representative IL-6 ELISA for Thp-1 supernatants after exposure to LPS with/ without rAOAH at increasing concentrations at pH 5 and pH 7.5. Detoxification of LPS was carried out by co-incubation with rAOAH in buffer conditions. Data is representative for three independent experiments, with each condition done in duplicates.

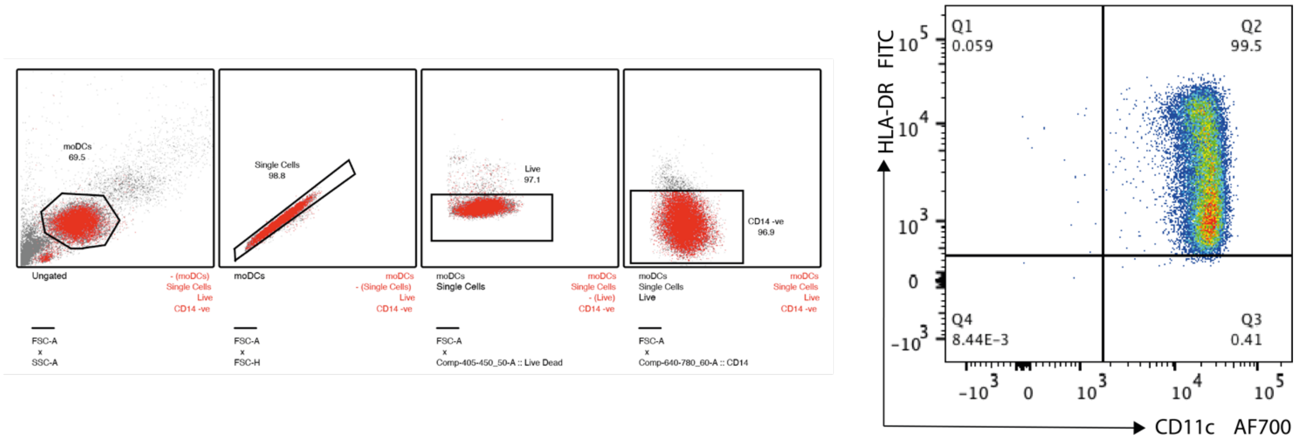

Figure S2.

Representative gating strategy used for monocyte-derived DCs and Langerhans cell-like cells. CD14- CD11c+ and HLA-DR+ cells were further analysed for cells surface markers CD1a and Langerin.

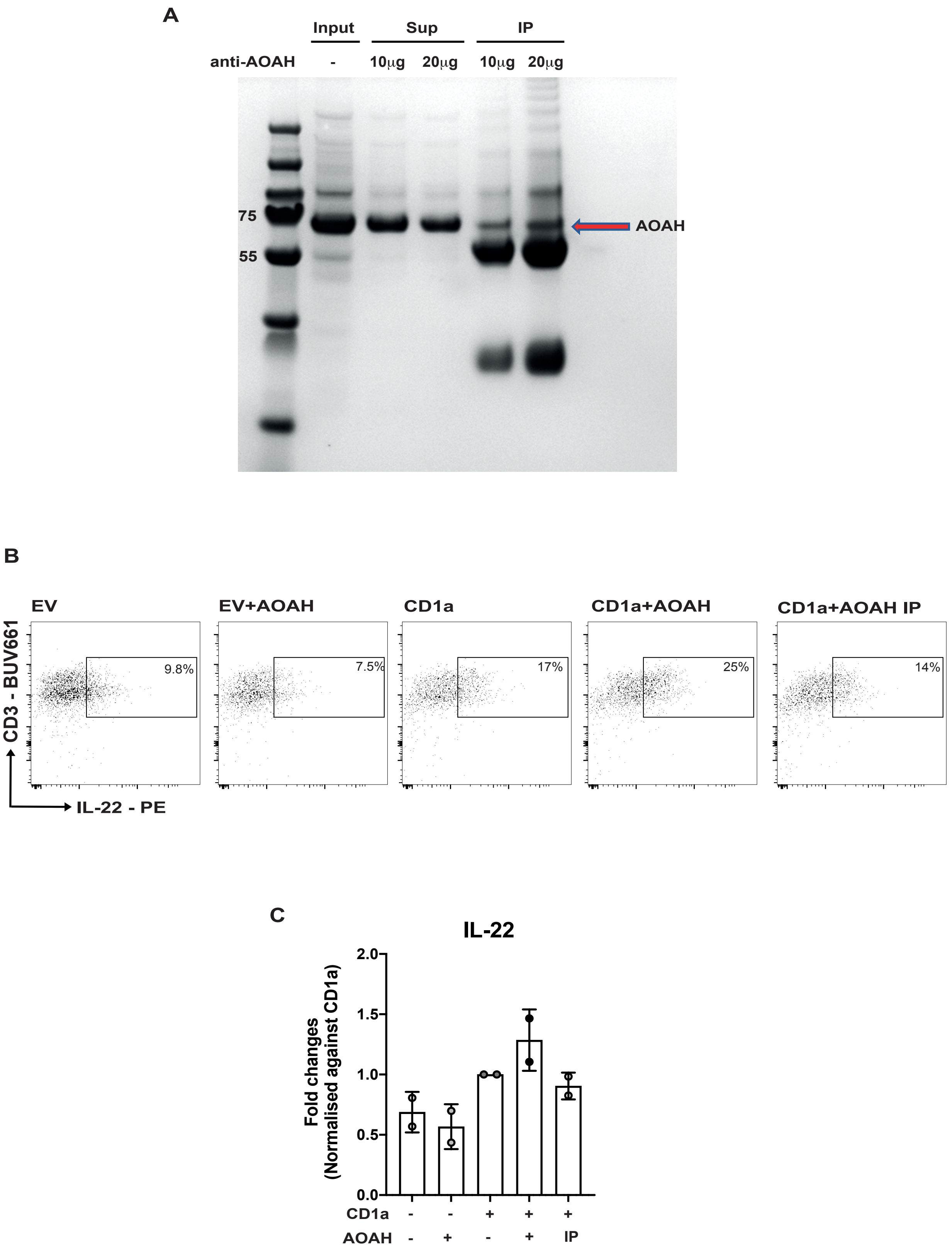

Figure S3.
Removing AOAH protein attenuated AOAH-specific IL-22 production by CD1a-autoreactive T cell clones. Representative Coomassie blue stained SDS-PAGE gel of AOAH protein after immunoprecipitation using anti-AOAH antibody. Input lane. Recombinant AOAH protein, Sup lane. Supernatant from the IP reaction, IP. Boiled beads containing anti-AOAH antibodies and immunoprecipitated AOAH. The detected bands for AOAH approximately matched the predicted size for AOAH at ~ 66 kDa (A). IL-22 production from each CD1a-reactive T cell clones after removing AOAH was determined by flow cytometry (n=2) (B-C). The data in A and B is representative of two independent experiments.

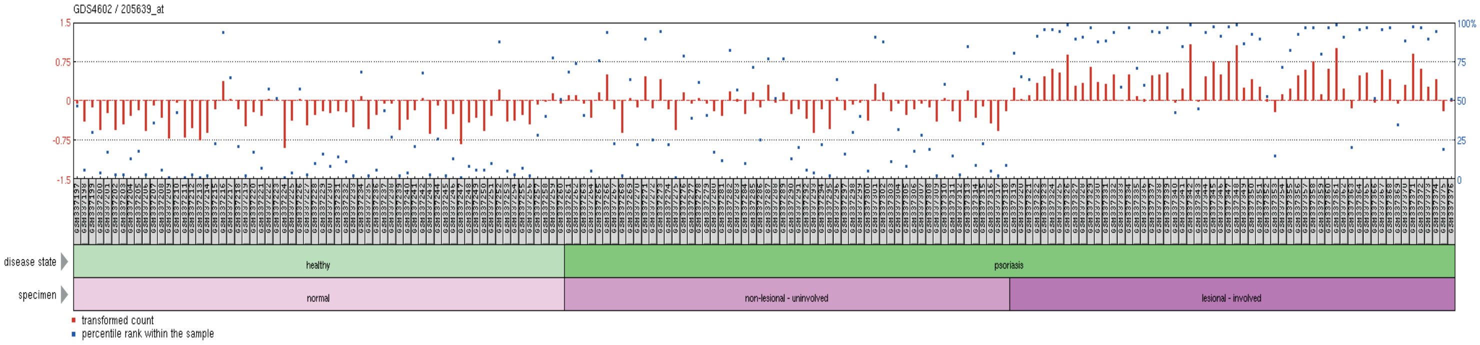

Figure S4.
Gudjonsson et al., 2009 performed microarray experiments on total RNA extracted from un-involved non-lesional and involved lesional skin from psoriasis patients and healthy skin. A search for AOAH in their data deposited in GEO profiles revealed AOAH to be up-regulated in lesional psoriatic skin compared to un-involved non-lesional skin from the same patient and healthy skin from healthy volunteers with no history of skin conditions (data accessible at NCBI GEO database (Gudjonsson et al., 2009) (accession GSE13355, https://www.ncbi.nlm.nih.gov/geoprofiles/100687287). Y-axis data expressed as arbitrary units.

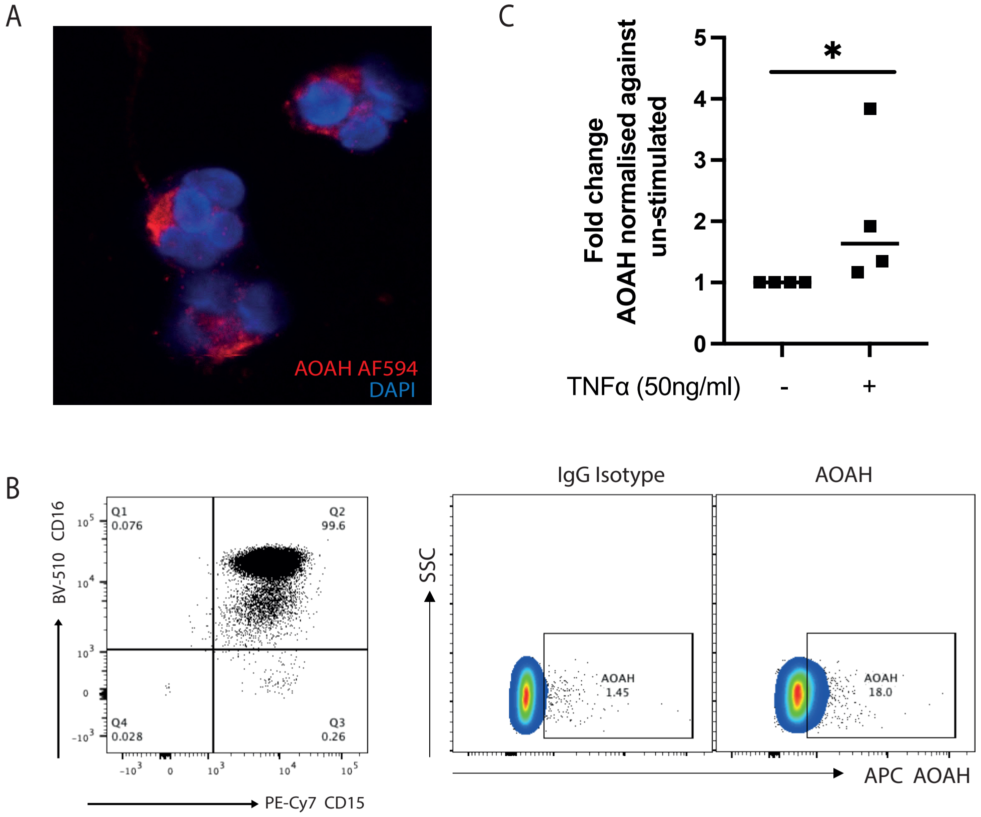

Figure S5.
AOAH constitutively expressed in unstimulated neutrophils and secreted upon TNF-α stimulation. Immunofluorescence staining of acetone fixed neutrophils from healthy controls. Cells were incubated with anti-human AOAH antibody with a secondary conjugated to AF 594 (A). Intracellular staining for AOAH in un-stimulated neutrophils from healthy controls compared to IgG isotype staining. Data are representative of 3 donors from 3 separate experiments (B). ELISA for AOAH on supernatants from neutrophils stimulated with 50 ng/ml of TNF-α for 18 hours. Data are representative of 4 donors from three separate experiments; non-parametric t-test was used to compare fold change between the two conditions (C). *P<0.05.
